# Efficacy of Elaeagnus Angustifolia extract in the treatment of knee osteoarthritis: a randomized controlled trial

**DOI:** 10.17179/excli2015-639

**Published:** 2016-03-02

**Authors:** Yunes Panahi, Gholam Hossein Alishiri, Noushin Bayat, Seyed Morteza Hosseini, Amirhossein Sahebkar

**Affiliations:** 1 Chemical Injuries Research Center, Baqiyatallah University of Medical Sciences, Tehran, Iran; 2 Department of Rheumatology, Baqiyatallah University of Medical Sciences, Tehran, Iran; 3 Biotechnology Research Center, Mashhad University of Medical Sciences, Mashhad, Iran; 4 Metabolic Research Centre, Royal Perth Hospital, School of Medicine and Pharmacology, University of Western Australia, Perth, Australia

**Keywords:** osteoarthritis, Elaeagnus angustifolia, pain, NSAID, clinical trial

## Abstract

Osteoarthritis (OA) is one of the most common musculoskeletal disorders all over the world. Available anti-arthritic medications have only partial efficacy and their long-term use is associated with adverse events. *Elaeagnus Angustifolia* (EA) is a medicinal plant with analgesic and anti-inflammatory properties. The present study evaluated the impact of two doses of EA extract compared with ibuprofen on the severity of disease in patients with knee OA. This study was designed as a randomized, double blind, active-controlled and parallel group trial. Patients with OA were randomized to receive 300 mg/day (n=33) or 600 mg/day (n=32) of EA aqueous extract, or 800 mg/day ibuprofen (n=32) for 7 weeks. EA extract contained 0.21 % (w/w) kaempferol according to HPLC. Efficacy of treatment was assessed using Western Ontario and McMaster Universities Osteoarthritis Index (WOMAC), Visual Analogue Scale (VAS) of pain, Lequesne's Pain-Function Index (LPFI), and patient's global assessment (PGA) index. The amount of kaempferol in the extract was determined by HPLC method to be 0.21 % w/w. There were significant reductions in WOMAC, VAS, LPFI and PGA scores by the end of trial with all three interventions. Comparison of the changes in WOMAC, VAS and LPFI scores among the treatment groups did not reveal any significant difference between EA and ibuprofen, and between low and high doses of EA. EA was safe and well tolerated during the course of trial and no adverse event was reported. The present results suggest beneficial effects of aqueous EA extract in reducing the symptoms of OA with an efficacy comparable to that of ibuprofen.

## Introduction

Osteoarthritis (OA) is the most common joint disease in adults, and its prevalence increases with age causing disability in the elderly population (Felson et al., 2000[11]). The most common symptoms of OA are pain, joint stiffness, crepitation on motion and limitation of joint motion (Hochberg et al., 1995[12]). Pain is a debilitating symptom of OA which causes significant impairment to the quality of life of afflicted patients. Routinely administered medications for OA include analgesics and non-steroidal anti-inflammatory drugs, but these drugs may introduce gastrointestinal and renal complications in long-term use (Smalley et al., 1995[24]; Felson et al., 2000[11]). Therefore, there is a demand for novel anti-nociceptive and anti-inflammatory medications that could control OA-associated chronic pain in an effective and safe fashion (Walzer et al., 2015[26]; Chen et al., 2015[8]; Panahi et al., 2014[20]; Sahebkar and Henrotin, 2015[22]).

Medicinal plants have always been regarded as an indispensible source for finding natural medicines with multi-faceted actions and minimal adverse events (Cameron et al., 2009[7]). The plants belonging to the genus *Elaeagnus* (Elaeagnaceae) grow widely over vast swathes of land from the Northern regions of Asia to the Himalayas and Europe. One species of this genus, Elaeagnus angustifolia (EA), also known as Russian olive, has been reported in the Iranian folk medicine to be used for its anti-inflammatory and analgesic properties in the treatment of patients with rheumatic diseases (Mirhydar, 1998[17]). Other conditions for which EA has been traditionally used as a medicine include fever, amoebic dysentery, gastrointestinal problems (nausea, vomiting and jaundice), jaundice, tetanus and asthma (Mirhydar, 1998[17]). Noteworthy, pharmacological investigations have revealed that EA extract has potent anti-inflammatory, analgesic and muscle relaxant effects (Ahmadiani et al., 2000[1]; Ramezani et al., 2001[21]; Hosseinzadeh et al., 2003[13]). Nevertheless, reports on the efficacy of EA in the management of OA in clinical practice have been very scarce (Ebrahimi et al., 2014[9]) and no previous head-to-head comparison with an NSAID exists. The present study set out to address this question in a randomized clinical trial controlled with ibuprofen as a standard medication routinely used for the management of OA symptoms.

## Methods

### Subjects

Patients aged 50 to 80 years with a diagnosis of knee OA according to the clinical and radiological criteria of the American College of Rheumatology and experiencing moderate to severe pain on active movement (minimum 40 mm on l00-mm Visual Analog Scale) (Kawasaki et al., 1998[15]; Wu et al., 2005[27]) were considered eligible for participation in the study. Exclusion criteria were:

1. concomitant osteoarticular disorders;
2. rheumatoid arthritis
3. gout
4. uncontrolled hypertension (blood pressure > 140/90 mm Hg)
5. New York Heart Association class III or IV heart failure
6. chronic renal failure (serum creatinine ≥ 1.5 mg/dL)
7. liver function impairment (any liver function test 1.5 times the upper limit of normal)
8. erythrocyte Sedimentation Rate > 20 (ESR)
9. history or current evidence of gastric and/or duodenal ulcer
10. impairment of motor function not due to OA of the hip or knee
11. confirmed hypersensitivity to the study drugs (including NSAID-induced Bronchoconstriction)
12. phenylketonuria
13. myocardial infarction
14. cerebrovascular event in the last 12 months
15. plans for prosthetic replacement of a hip or knee in the next 6 months
16. lack of compliance
17. treatment with NSAIDs during the last week, or with corticosteroids during the last 4 months (including intra-articular corticosteroids)
18. need for physical treatment (e.g. mobilization) during the first 2 weeks of the study; and
19. treatment with furosemide, probenecid, anticoagulants, hydantoin, sulfonamides, lithium salts, methotrexate, beta-blockers, or muscle relaxants.

### Study design

This study was designed as a pilot randomized, double-blind, active-controlled and parallel group phase II clinical trial. Patients were selected from those referring to the Baqiyatallah Hospital (Tehran, Iran), and were randomized in a 1:1:1 ratio to receive low (300 mg/day)-dose EA extract (n=33), high (600 mg/day)-dose EA extract (n=33), or ibuprofen at a dose of 800 mg/day (n=33) for a period of 7 weeks. Selection of dose was based on previous studies showing the efficacy and safety of EA products (Alishiri et al., 2007[2]; Ebrahimi et al., 2014[9]; Nikniaz et al., 2015[18]). EA and ibuprofen were administered as syrup, and filled in bottles identical in shape and size. Patients were advised to use their administered medication in two divided doses. The syrups were coded following a computer-generated randomization list. Each bottle bore a label with a number corresponding to a patient specified by that number, and was coded with the study drug allocated to that patient according to the randomization list. The subjects were allocated a randomization number in consecutive order and were given the corresponding drug supplies. This study was approved by the institutional Ethics Committee and written informed consent was obtained from all participants.

### Efficacy measures

The variables for the assessment of efficacy were changes in the Western Ontario and McMaster Universities Osteoarthritis (WOMAC) index, Visual Analogue Scale (VAS) of pain, Lequesne's Pain-Function Index (LPFI), and Patient's Global Assessment (PGA) Index. The variables for the assessment of tolerability included incidence of all adverse reactions including gastrointestinal complications, and abnormal changes in vital signs, routine hematological and biochemical tests including erythrocyte count, white blood cell count (WBC) and differential, uric acid, blood urea nitrogen (BUN), serum creatinine, aminotransferases and alkaline phosphatase. 

### Assessments

WOMAC index was used for the assessment of OA symptoms. WOMAC subscales include pain (5 items), stiffness (2 items) and physical functioning (17 items). Each item is scored from 0 to 4, yielding a score range of 0-20, 0-8 and 0-68 for pain, stiffness and physical functioning subscales, respectively. Several studies have approved the reliability and validity of WOMAC index (Bellamy et al., 1988[4]; Basaran et al., 2010[3]).

PGA was rated according to a 4-point semi-quantitative rating scale: 3 for excellent, 2 for good, 1 for moderate, and 0 for insufficient control of OA symptoms. To assess VAS score, a 100 mm rating scale was designed with a range from ''no problem whatsoever'' (score 0) to ''unbearable'' (score 100). Patients were instructed to mark a place on horizontal line reflecting their self-perceived health status.

LPFI index consists of 3 subscales with a total of 10 items. The pain or discomfort scale has 5 items, the maximum distance walked has 1 item, and the function or activities of daily living (ADL) has 4 items. The pain and ADL scales have a score range from 0 (reflecting no pain or functional limitation) to 8 (reflecting extreme pain or functional limitation). The “maximum distance walked” have a range from 0 (unlimited) to 6 (less than 100 m). The score is increased by 1 point “if the patient uses one walking stick or crutch”, or by 2 points 'if the patient uses two walking sticks or crutches”. Total Lequesne index ranges from 0 to 24, with higher scores exhibiting worse health status (Nikniaz et al., 2014[19]). 

### Plant material

EA fruits were collected from the Jolfa region in the West Azerbaijan province (Northwest of Iran). Plant was identified and stored in the herbarium of the Pharmacognosy Laboratory at the Shaheed Beheshti University of Medical Sciences (Voucher numbers 96-110).

### Extraction procedure

The whole fruits and their individual components were milled. Two hundred and fifty grams of milled fruit were added to 1000 mL of boiling water and the mixture was boiled for 20 minutes before being filtered through a two-layer mesh. The water extract was concentrated on a boiling bath to the desired concentration before being cooled and stored in a freezer at -20 °C. Moisture of extract was determined as follows: 2 g of final extract was placed in an oven in 60 °C for 72 h, and then weighed, and the loss of weight was then used as a moisture indicator. The final extract contained 25 % water. The extract was dissolved in distilled water at the desired concentration to make the syrup.

### Determination of kaempferol content of the EA extract

Dried aqueous extract (1 g) was dissolved in water (10 mL) and 25 % hydrochloric acid (10 mL), and heated in a water bath at 83 °C for 120 min. After removing solvent by a rotary evaporator, the residue was dissolved in methanol (10 mL) and filtered through a filter paper (0.4 µm). A high-performance liquid chromatography (HPLC) method was developed in a reverse-phase condition to assay the kaempferol content in the filtered extract. A C18 column was used with a UV detector to assay kaempferol at 365 nm (λmax). The mobile phase was 1M H_3_PO_4_, acetonitrile, water and methanol (1:10:30:60) with a pH adjusted to 4. The flow rate was 1.4 mL/min. Rutin (0.05 mg/ mL) was added to samples as an internal standard. The injection volume was 30 µL. To determine the amount of kaemferol in the extract, a calibration curve was constructed. Different concentrations of kaemferol (0.25, 0.41, 0.6, 0.8, 1.1, 1.65 and 2 ng/mL in methanol) were assayed to plot the standard curve. Each HPLC measurement was performed in triplicate.

### Statistical analysis

All statistical procedures were performed using SPSS version 16 software (SPSS International BV, Chicago, IL). Within- and between-group comparisons were performed using paired samples *t*-test and one-way ANOVA, respectively. In all comparisons, a two-sided *p*-value of < 0.05 was considered as statistically significant.

## Results

**Figure 1:**
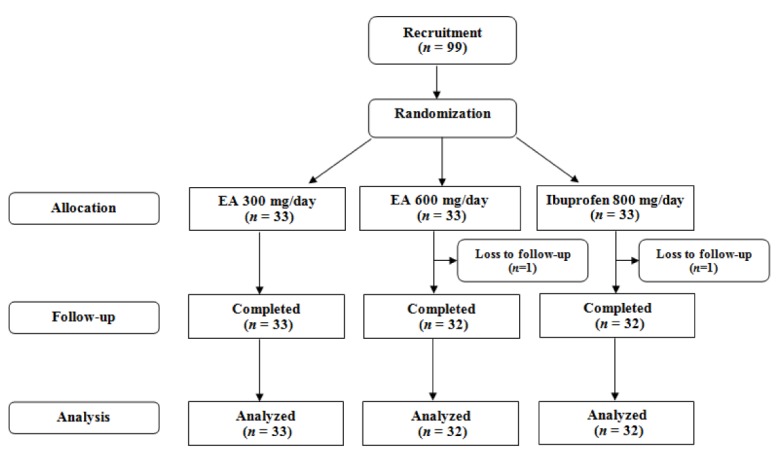
Flow chart of the trial

**Figure 2:**
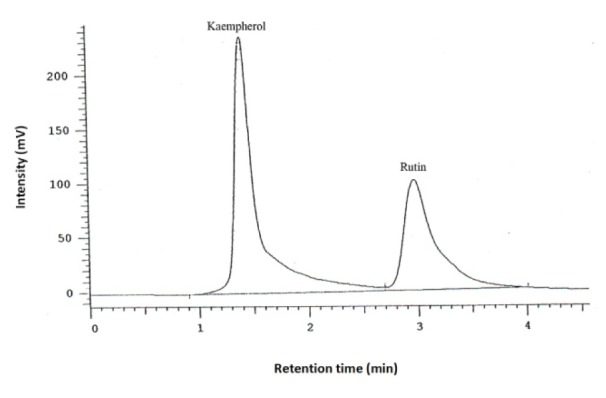
HPLC determination of kaempferol content in the *Elaeagnus angustifolia *extract. Rutin was used as the internal standard

Of the initial 99 patients who met the inclusion criteria, 97 (97.9 %) finished the study comprising 33 in the low-dose EA (300 mg/day), 32 in the high-dose EA (600 mg/day) and 32 in the ibuprofen group (Figure 1(Fig. 1)). There was no significant difference in age and gender, as well as baseline BMI, VAS, LPFI, WOMAC and PGA scores among the three studied groups (*p *> 0.05) (Table 1(Tab. 1)). The amount of kaempferol in the extract was determined by HPLC method to be 0.21 % w/w (Figure 2(Fig. 2)).

There were significant reductions in the total WOMAC score following 7-week treatment in all three studied groups. With respect to subscales, there were significant reductions in the pain and stiffness subscales in the high-dose (600 mg/day) (p=0.028) and low-dose (300 mg/day) (p=0.035) *E.* *angustifolia* group, respectively. Physical function was improved in the ibuprofen (p=0.038) and high-dose *E.* *angustifolia *(p=0.009) groups. VAS, LPFI and PGA scores were all improved by either of administered EA doses and ibuprofen (Table 2(Tab. 2)).

Between-group comparisons indicated that the changes in total and subscale WOMAC, VAS and LPFI scores were comparable among the three studied groups. However, there was a greater reduction in the PGA score in the low-dose *E.* *angustifolia* group compared with the ibuprofen group (p=0.048), whilst the difference with the high-dose vs. low-dose *E.* *angustifolia* groups, and between the high-dose and ibuprofen groups did not reach statistical significance (Table 3(Tab. 3)).

No significant differences were seen in adverse reactions or tolerability between the study groups. No significant gastrointestinal adverse reactions were seen in the patients treated in the three treatment groups.

## Discussion

The goal of this study was to evaluate the efficacy of *E. angustifolia *extract in the treatment of knee osteoarthritis pain and clinical symptoms. The results showed a significant decrease in pain and other symptoms of osteoarthritis by *E. angustifolia *extract, with an efficacy comparable to that of ibuprofen as a standard analgesic.

Although there are many studies conducted on the efficacy of *E. angustifolia* in gastrointestinal disorders, few studies have assessed the analgesic and anti-inflammatory effects of this medicinal plant in clinical settings. Ebrahimi et al. (2014[9]) reported significant improvements in WOMAC total score as well as subscales of pain and physical function following 8 weeks of supplementation with *E. angustifolia* medulla powder in females with knee osteoarthritis. However, no improvement versus placebo was observed with the whole fruit powder (Ebrahimi et al., 2014[9]). In the same study, measurement of circulating levels of a number of cytokines including tumor necrosis factor-alpha (TNF-α), interleukine-1β, interleukine-10, matrix metalloproteinase-1 and -13 did not suggest any significant change compared with placebo (Nikniaz et al., 2014[19]).

Previous experimental studies have shown that different constituents of *E. angustifolia *seeds possess anti-nociceptive activity in hot-plate and writhing tests, therefore suggesting the involvement of both peripheral and central nervous mechanisms (Ahmadiani et al., 2000[1]; Ramezani et al., 2001[21]). Also, it has been reported that *E. angustifolia* can suppress both cyclooxygenase-1 and -2 enzymes with a comparable efficacy to indomethacin (Farahbakhsh et al., 2011[10]). This could be an important mechanism for the efficacy of the plant in OA, as cyclooxygenase enzymes are responsible for the production of a range of pro-inflammatory and nociceptive mediators, particularly prostaglandin E_2_ (PGE_2_) (Bensen et al., 1999[5]).

The aqueous and ethanol extracts of *E. angustifolia* have a high content of flavonoids and tannins. Kaempferol, ferulic acid and cumaric acid are the major polyphenolic components of *E. angustifolia *extract (Bucur et al., 2009[6]). The action of kaempferol is of particular interest owing to the efficacy of this flavonoid in suppressing the production and release of inflammatory cytokines (TNF-α and interleukin-6), mediators (nitric oxide and PGE_2_) and signaling molecules (Src, Syk and IRAK4), and release of reactive oxygen species (Kim et al., 2015[16]).

Aqueous and ethanol extracts of the plant have been reported to possess muscle-relaxant activity in a dose-dependent manner. Interestingly, this effect of *E. angustifolia* (at the dose of 2 mg/kg) was comparable to that of diazepam (Hosseinzadeh et al., 2003[13]). Muscle-relaxant effect of the extract can be a potential mechanism for the pain-relieving effects of *E. angustifolia* in patients with OA as peri-articular muscles weakness is common in this disease (Hurley, 1999[14]). However, muscle-relaxant effects may be harmful in long-term because strengthening the quadriceps muscles is required for the treatment of OA (Serrão et al., 2012[23]; van der Esch et al., 2012[25]). Hence, it is required to evaluate muscle torque changes after treatment with *E. angustifolia* to confirm the mentioned possible side-effect.

In conclusion, findings of the present trial suggested the efficacy and safety of *E. angustifolia* extract in alleviating the clinical symptoms of OA. Although the present study was limited by its pilot nature, the results supported comparable efficacy of *E. angustifolia* with ibuprofen that was observed in this study is of particular importance owing to the side effects associated with the long-term use of NSAIDs. According to the present results, *E. angustifolia *extract could be regarded as an alternative to NSAIDs in subjects suffering from OA symptoms.

## Acknowledgement

The authors would like to thank Clinical Trial Research Center (Tehran, Iran) for the financial support of this study.

## Conflict of interest

None declared.

## Figures and Tables

**Table 1:**
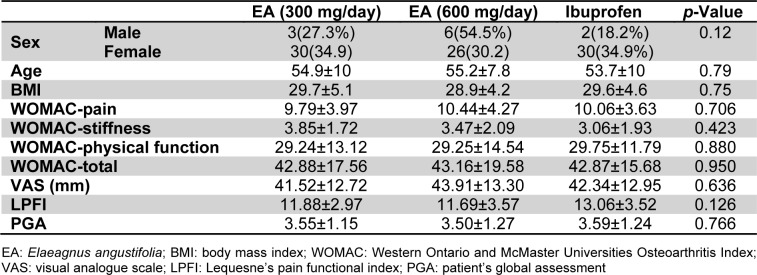
Demographic characteristics of the study groups

**Table 2:**
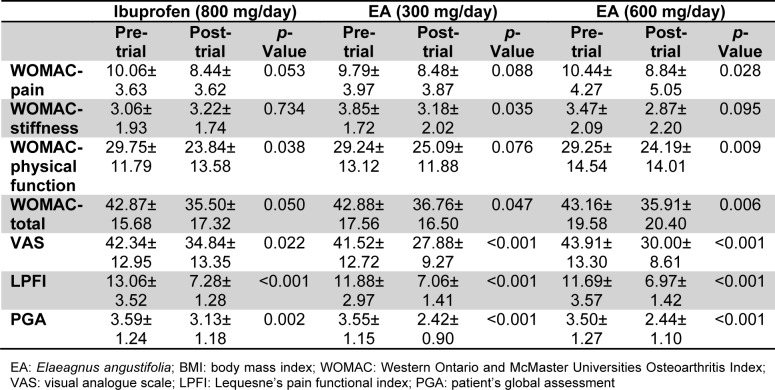
Changes in the efficacy measures within the study groups

**Table 3:**
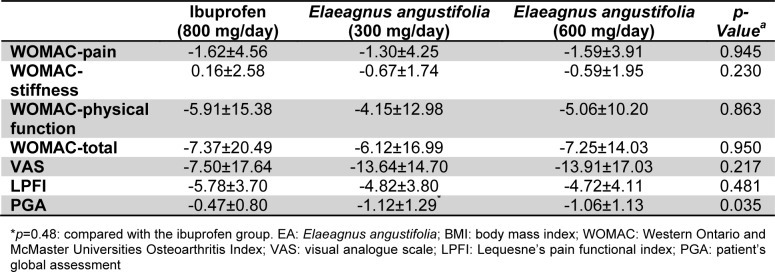
Comparison of changes in the efficacy measure between the study groups
